# A Photonic Crystal Magnetic Field Sensor Using a Shoulder-Coupled Resonant Cavity Infiltrated with Magnetic Fluid

**DOI:** 10.3390/s16122157

**Published:** 2016-12-16

**Authors:** Delong Su, Shengli Pu, Lianmin Mao, Zhaofang Wang, Kai Qian

**Affiliations:** 1 College of Sciene, University of Shanghai for Science and Technology, Shanghai 200093, China; 142231886@st.usst.edu.cn (D.S.); 142231880@st.usst.edu.cn (L.M.); 142231887@st.usst.edu.cn (Z.W.); 2 Shanghai Key Laboratory of Modern Optical System, University of Shanghai for Science and Technology, Shanghai 200093, China; 3 School of Information Engineering, Hubei University for Nationalities, Enshi 445000, Hubei Province, China; qiankai0565@163.com

**Keywords:** magnetic fluid, photonic crystal, resonant cavity, magnetic field sensor

## Abstract

A kind of photonic crystal magnetic field sensor is proposed and investigated numerically. The shoulder-coupled resonant cavity is introduced in the photonic crystal, which is infiltrated with magnetic fluid. Through monitoring the shift of resonant wavelength, the magnetic field sensing is realized. According to the designed infiltration schemes, both the magnetic field sensitivity and full width at half maximum increase with the number of infiltrated air holes. The figure of merit of the structure is defined to evaluate the sensing performance comprehensively. The best structure corresponding to the optimal infiltration scheme with eight air holes infiltrated with magnetic fluid is obtained.

## 1. Introduction

Magnetic fluid (MF) is a kind of stable colloidal solution consisting of surfactant-coated nanoscale magnetic particles [1]. It has both the magnetic properties of magnetic materials and the fluidity of liquids, which shows a variety of magneto-optical characteristics including Faraday rotation, tunable refractive index, and magneto-birefringence [2,3,4,5]. Many optical devices based on MF have been proposed in the past decades [6,7,8,9,10,11,12]. Compared with the traditional devices, MF-based optical devices have the advantages of high sensitivity and a small size, which makes them promising for broad applications in the fields of photonics and sensing [13,14,15,16,17,18,19].

In 2014, Yang et al. proposed and experimentally demonstrated a magnetic field sensor based on the tilted fiber Bragg grating coated with MF and found that the maximum resonant wavelength shift can reach 106 pm at a magnetic field strength of 32 mT [20]. In 2015, Liu et al. put forward an optical magnetic field sensor with temperature compensation capability, which is based on an optical microfiber taper combined with MF [21]. Its corresponding sensitivity is 0.171 nm/Oe in the range of 20–70 Oe at 25 °C. In 2015, Pu et al. proposed and experimentally demonstrated a late-model magnetic field sensor based on a microfiber coupler surrounded with MF, which also has the potential applications for designing other tunable all-in-fiber photonic devices, such as the magneto-optical modulator and filter [22].

It is well-known that light with a frequency lying within the photonic band gap can be guided or spatially localized by the photonic crystal (PC) structure with certain line or point defects [23,24]. PC waveguides combined with novel materials (i.e., magnetic materials and graphene) have been proposed for tuning the slow light [25,26,27,28], which refers to light with a low group velocity, and is expected to become instrumental in enabling applications in quantum computing, ultrafast all-optical information processing, and so on. Meanwhile, because of the high quality factor [29], the PC cavity is a good candidate for realizing narrow-bandwidth filters [30], optical switches [31], cavity quantum electrodynamics [32], and sensors [33,34,35]. The resonant wavelength of the PC cavity is highly sensitive to the effective refractive index (RI) of the cavity defect. When the RI of the PC cavity varies slightly, a measurable resonant wavelength shift can be detected [36,37]. In this work, a kind of PC magnetic field sensor based on shoulder-coupled cavity infiltrated with MF is proposed. Compared to other cavity coupling configurations, the shoulder-coupled cavity can have a stronger coupling strength [38], which is desirable for detecting the shift of the resonant wavelength in transmittance spectra.

## 2. Device Configuration and Sensing Principle

### 2.1. Device Configuration

**Figure 1:**
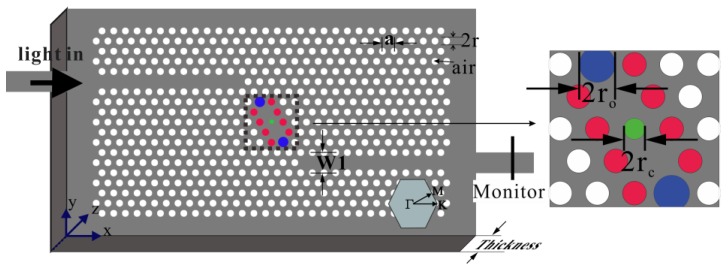
Schematic of the sensor configuration.

Figure 1 is the schematic of the silicon slab PC structure with shoulder-coupled cavity. The aslant shoulder-coupler cavity in between the two W1 waveguides is formed by removing two air holes adjacent to the central air hole along the oblique direction. The sizes of the center air hole and two terminal air holes along the aslant cavity (marked in green and blue in Figure 1) are optimized. The center air hole and the air holes around the removed ones (marked in green, blue, and red in Figure 1) are infiltrated with MFs according to various infiltration schemes. The lattice constant a = 423 nm, the radius of normal air holes is r = 0.32a, and the thickness of the PC slab is h = 0.55a. The RI of silicon is n_Si_ = 3.48 at λ = 1550 nm. Light is injected into the shoulder-coupled cavity through the input W1 waveguide, and the leaky light from the cavity is monitored at the end of the output W1 waveguide.

### 2.2. Sensing Principle

Due to the waveguide-microcavity coupling, the resonant wavelengths will appear as peaks in the transmission spectrum. When the magnetic field is applied, the RI of the MF will change, as does the effective RI of the MF-infiltrated cavity. To be general, the local magnetic field factor $\alpha_{//}$ is employed to express the magnetic field qualitatively [39,40,41]. The value of $\alpha_{//}$ lies in the range of $0<\alpha_{//}<1$ and is inversely proportional to the strength of the externally applied magnetic field. In other words, $\alpha_{//}\;=\;1$ corresponds to a zero magnetic field, while $\alpha_{//}\;=\;0$ indicates an infinite magnetic field. Thus, the resonant wavelength of the structure is related with ${\lambda \;=\;k}_{\alpha_{//}}\times n(\alpha_{//})$, where $k_{\alpha_{//}}$ is a constant, and $n(\alpha_{//})$ is the magnetic-field-dependent effective RI of the MF-infiltrated cavity. Thus, through monitoring the resonant wavelength shift of the shoulder-coupled cavity, the externally magnetic field can be measured. For numerical simulation, the MF RIs at different local magnetic field factors $\alpha_{//}$ (corresponding to different magnetic field strengths) in [27] is utilized.

## 3. Modeling Methodology and Structure Optimization

### 3.1. Modeling Methodology

The finite-difference time-domain (FDTD) method [42,43] is used to investigate the sensing structure numerically. The type of mesh generation is non-uniform automatically. The number of mesh points per wavelength (ppw) is set at 22 in this work, i.e., each grid is divided into 22 × 22 points per wavelength. The perfectly matched layer (PML) is used. The 2D effective index method instead of 3D calculation is employed. The variational method [43] is utilized to calculate the 2D effective index, which depends on the type and polarization of the source, the distribution of the vertical index, the thickness of the slab, and the central wavelength. The 2D effective index is obtained to be 2.86 for the TE-like fundamental mode of the 1550 nm source.

### 3.2. Optimization of Shoulder-Coupled Cavity

**Figure 2:**
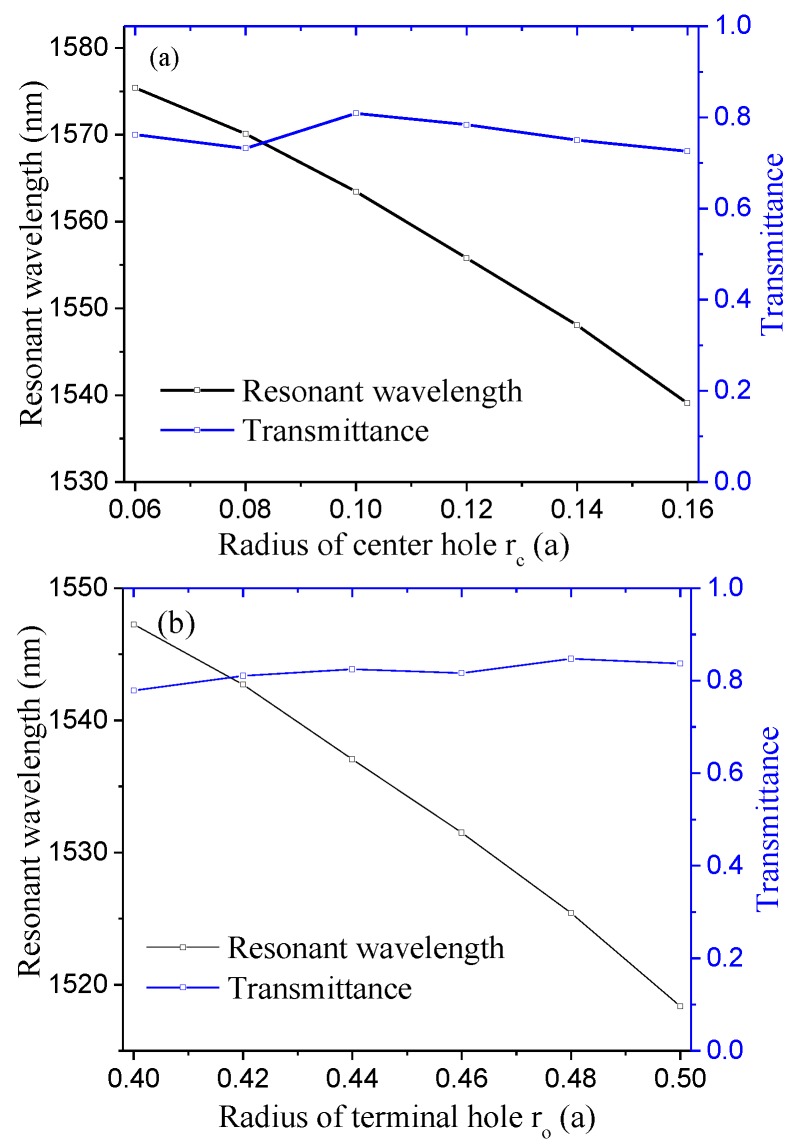

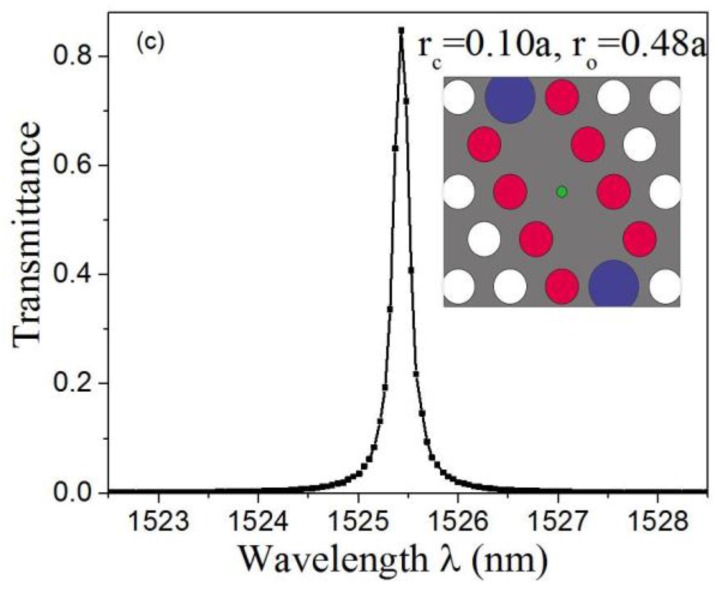
(**a**) Resonant wavelength and peak transmittance as functions of the radius of the center hole when $r_{o}\;=\;0.32a$; (**b**) the radius of the terminal hole when $r_{c}\;=\;0.10a$; (**c**) the transmission of the TE-like fundamental mode for $r_{c}\;=\;0.10a$; and $r_{o}=0.48a$.

For the coupling between the W1 waveguide and the aslant cavity, the cavity structural parameter along the light path is most crucial. Therefore, the radiuses of the center hole (r_c_) and terminal holes (r_o_) of the aslant cavity (marked in green and blue in Figure 1) are chosen to optimize the resonant transmission. To optimize the radius of center hole r_c_, r_c_ varies from 0.06a to 0.16a with an increment of 0.02a (r_o_ is fixed at 0.32a, i.e., same as the normal air hole). The corresponding resonant wavelength and peak transmittance as functions of the radius of the center hole is shown in Figure 2a. Figure 2a indicates that the transmission is maximum for $r_{c}\;=\;0.10a$. Therefore, $r_{c}\;=\;0.10a$ is chosen to optimize the radius of terminal hole r_o_. r_o_ varies from 0.40a to 0.50a with an increment of 0.02a. The corresponding resonant wavelength and peak transmittance as functions of the radius of the terminal hole is shown in Figure 2b. Figure 2b indicates that the transmission is maximum for $r_{o}\;=\;0.48a$. For both optimization processes, the resonance is pushed to a higher frequency (blue-shift) with the increase in the hole radius. At the optimized structure parameters of $r_{c}\;=\;0.10a$ and $r_{o}\;=\;0.48a$, the distinctive transmission peak at a wavelength of 1525.43 nm is plotted in Figure 2c. The full width at half maximum (FWHM) of the transmission peak is 0.20 nm. The quality factor defined as ${Q\;=\;\lambda }_{0}/\mathrm{FWHM}$ (λ_0_ is the peak wavelength) is 7602, which is larger than those of other similar PC sensors [44,45,46]. The quality factors for the sensors in [44,45,46] are reported to be 400, 3000, and 2966, respectively.

**Figure 3:**
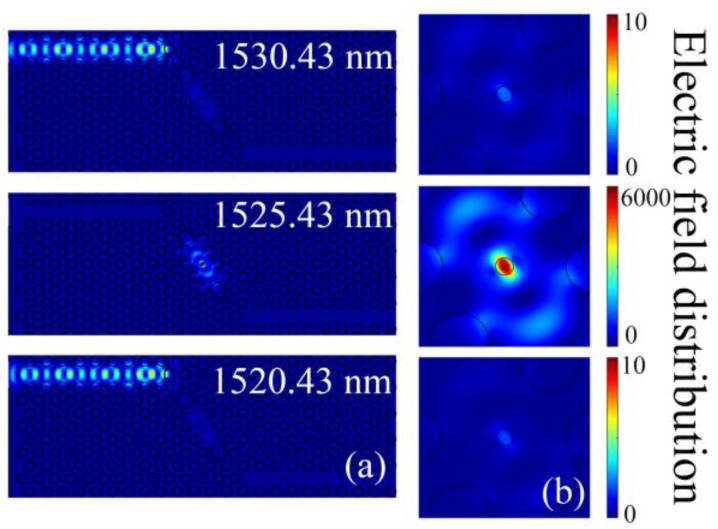
Steady-state electric field distribution for incident wavelengths of 1530.43, 1525.43 and 1520.43 nm. (**b**) is the cavity area corresponding to (**a**).

For the optimized structure, the steady-state field distribution of light propagation at wavelengths of 1520.43, 1525.43 and 1530.43 nm are simulated and plotted in Figure 3. Figure 3 displays the on-resonance state happens for incident wavelength of 1525.43 nm, while the cases for incident wavelengths of 1520.43 and 1530.43 nm are off-resonance states (noticing the great difference of color bar between the top/bottom and middle panels). The electric field intensity within the shoulder-coupled cavity at the on-resonance state is about 2500 times larger than that at the off-resonance states. For the off-resonance states, most of the incident light is reflected from the PC cavity, as can be seen in Figure 3a.

## 4. Sensing Results and Discussion

**Figure 4:**
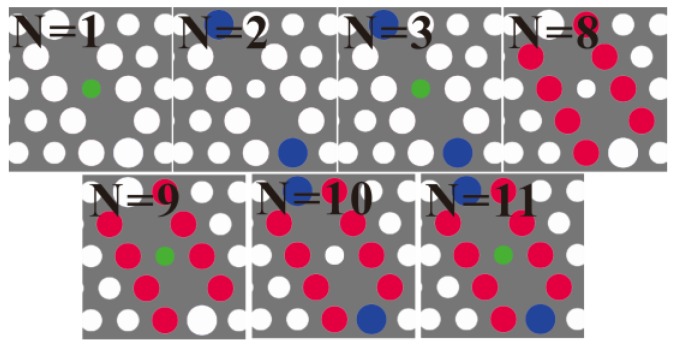
Various infiltration schemes for the structues with a shoulder-coupled cavity infiltrated with magnetic fluid (MF).

**Figure 5:**
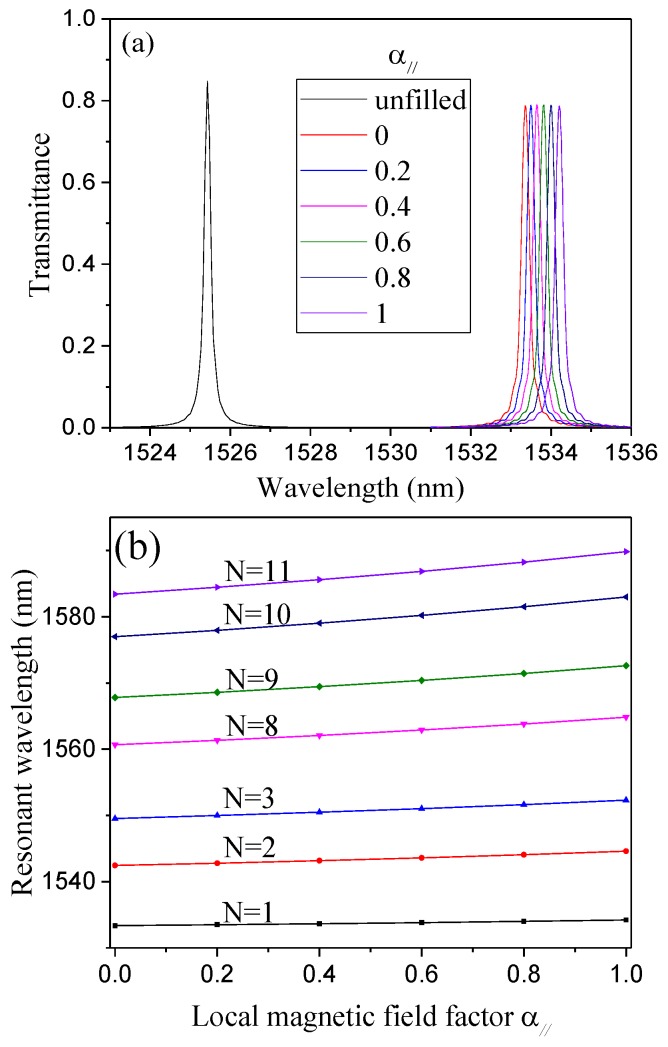
(**a**) Output transmission spectra at different local magnetic field factors $\alpha_{//}$ for the center air hole infiltrated with MF; (**b**) resonant wavelength shift with local magnetic field factor $\alpha_{//}$ for different MF-infiltrated structures.

The peak transmission wavelength-shift with magnetic field factor for various MF-infiltrated structures are investigated systematically. The infiltration schemes are illustrated in Figure 4. The calculated output transmission spectrum at different magnetic field factors $\alpha_{//}$ (viz. the RI of the MF ranging from 1.8787 to 1.9879) when only the center air hole is infiltrated with MF is shown in Figure 5a. Figure 5a reveals that the position of the resonant wavelength shifts towards a long wavelength as the magnetic field factor increases (i.e., the magnetic field strength decreases).

**Figure 6:**
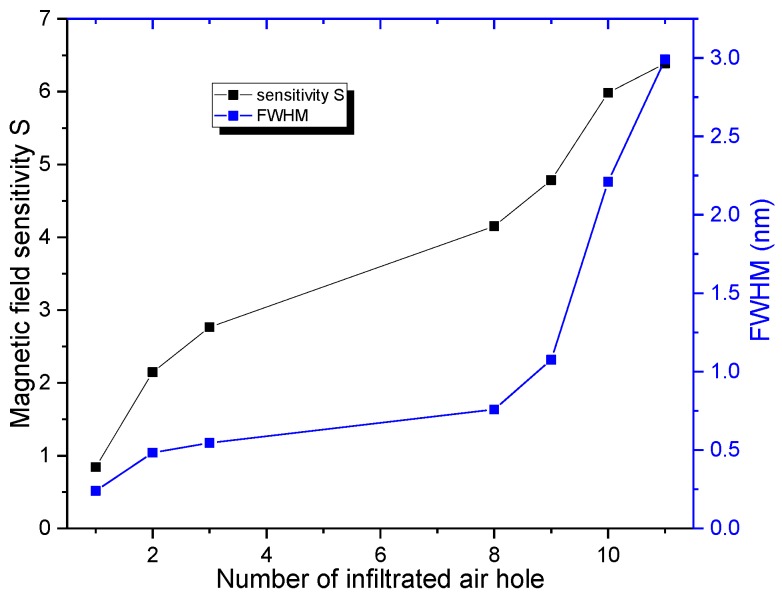
Magnetic field sensitiviy S and FWHM as functions of the number of infiltrated air holes.

**Figure 7:**
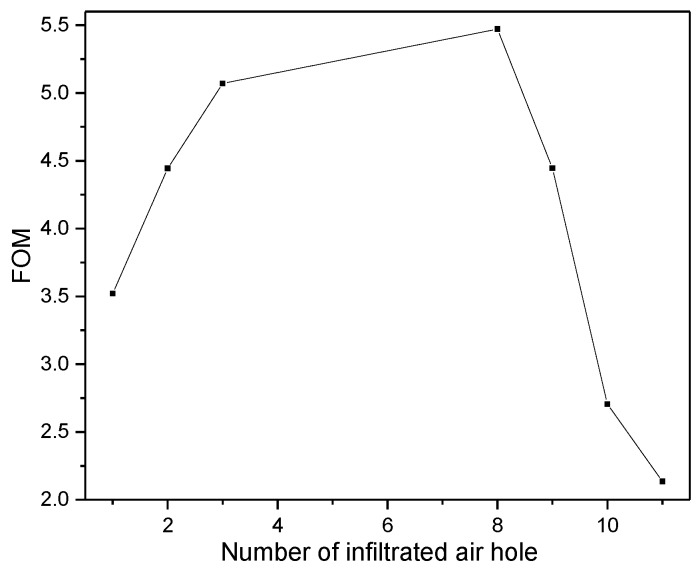
Figure of merit (FOM) of the infiltrated structure as a function of the number of infiltraed air holes.

Similarly, all other structures are simulated, and the corresponding results are shown in Figure 5b. Figure 5b shows that the resonant wavelength changes with the local magnetic field factor $\alpha_{//}$ linearly for all cases. In order to investigate the effect of infiltration schemes on the magnetic field sensitivity ($S\;=\;d\lambda_{0}/d\alpha_{//}$), the magnetic field sensitivity S (corresponding to the slopes of the curves in Figure 5b) as a function of the number of infiltrated air holes (N = 1, 2, 3, 8, 9, 10 and 11) is explicitly plotted in Figure 6. The corresponding FWHM is also plotted in Figure 6. Figure 6 shows that the magnetic field sensitivity S increases remarkably with the number of infiltrated air holes. In addition, the FWHM varies from 0.239 to 2.991 nm with the number of infiltrated air holes. For the pragmatic applications, the higher the magnetic field sensitivity is and the narrower the FWHM is, the better the sensing performance of the structure is. Therefore, there is a trade-off between the magnetic field sensitivity S and FWHM. The figure of merit (FOM) of the structures defined as FOM = S/FWHM is employed to evaluate the sensing performance comprehensively [47,48]. The corresponding results are shown in Figure 7. Figure 7 indicates that the FOM is maximum for the structure with N = 8. Therefore, the infiltration scheme corresponding to N = 8 (see Figure 4) is optimal.

## 5. Conclusions

A late-model silicon slab PC magnetic field sensor is proposed here. The shoulder-coupled cavity is designed for MF infiltration. The structure is optimized to obtain better sensing performance. Different MF infiltration schemes are investigated systematically. In view of sensing applications, the trade-off between the magnetic field sensitivity and FWHM is found for varying the number of infiltrated air hole according to the designed infiltrated schemes. Comprehensively, the optimum FOM of the sensing structures is found for the infiltration scheme with N = 8. The advantage of the proposed magnetic field sensor lies in its small size and potentiality for integrated devices. Besides, the structure of the PC cavity can allow for an even larger degree of multiplexing on monolithic substrates, which is an inherent advantage for optical integrated circuits, integrated optical devices, and monolithic integration.
